# Validity and reliability of a french version of the olfactory disorders questionnaire

**DOI:** 10.1186/s40463-022-00598-2

**Published:** 2022-10-01

**Authors:** Jérôme R. Lechien, Luigi A. Vaira, Serge D. Le Bon, Roxane Geerts, Paolo Boscolo-Rizzo, Sven Saussez

**Affiliations:** 1 Department of Otolaryngology - Head and Neck Surgery, EpiCURA Hospital, Baudour, Belgium; 2 Department of Human Anatomy and Experimental Oncology, Faculty of Medicine, UMONS Research Institute for Health Sciences and Technology, University of Mons (UMons), Mons, Belgium; 3 Department of Otolaryngology, Elsan Hospital, Paris, France; 4 Department of Otorhinolaryngology and Head and Neck Surgery, School of Medicine, Foch Hospital, UFR Simone Veil, Université Versailles Saint-Quentin-en-Yvelines (Paris Saclay University), Paris, France; 5 Department of Otolaryngology - Head and Neck Surgery, CHU Saint-Pierre, Brussels, Belgium; 6 Maxillofacial Surgery Operative Unit, Department of Medical, Surgical and Experimental Sciences, University of Sassari, Sassari, Italy; 7 Biomedical Science Department, PhD School of Biomedical Science, University of Sassari, Sassari, Italy; 8 Section of Otolaryngology, Department of Medicine, Surgery and Health Sciences, University of Trieste, Trieste, Italy; 9 Department of Otorhinolaryngology and Head and Neck Surgery, Polyclinic of Poitiers, Poitiers, France

**Keywords:** Olfactory, Smell, Olfaction, Anosmia, Odor, Rhinology, Head neck surgery, Otolaryngology

## Abstract

**Objective:**

To validate a French version of the Olfactory Disorders Questionnaire (Fr-ODQ).

**Methods:**

Patients with olfactory disorder (OD) and controls were enrolled from two medical centers. Individuals completed the Fr-ODQ and the French version of the sinonasal outcome tool-22 (SNOT-22). The extended Sniffin'Sticks procedure was used to test odor Threshold, Discrimination, and Identification (TDI). *Cronbach’s alpha* was used to measure the internal consistency of Fr-ODQ. The reliability and the external validity were evaluated through a test–retest approach and by correlating Fr-ODQ and SNOT-22 scores.

**Results:**

Eighty-nine patients with OD and 65 healthy individuals completed the evaluations. The Cronbach’s alpha was 0.827, reporting adequate internal consistency. The test–retest reliability was high (*r*_s_ = 0.944, *p* = 0.001). The external validity was adequate regarding the significant correlation between Fr-ODQ and SNOT-22 (*r*_s_ = 0.498; *p* = 0.001). Patients with OD reported a significant higher score of Fr-ODQ than healthy individuals (*p* < 0.001), indicating a high internal validity. The baseline Fr-ODQ significantly improved after 3-month olfactory training, which corroborated the improvement of TDI scores.

**Conclusion:**

The Fr-ODQ is the first patient-reported outcome questionnaire validated for French speaking patients. Fr-ODQ is reliable and valid for the evaluation of the olfactory dysfunction and the related impact on quality of life of French-speaking patients.

**Supplementary Information:**

The online version contains supplementary material available at 10.1186/s40463-022-00598-2.

## Introduction

Olfactory Dysfunction (OD) may affect 1 to 20% of the general population [1]. The primary causes of OD are sinonasal disorders, post-viral olfactory dysfunction, neurological diseases and post-traumatic lesions of the olfactory nerve [1]. The prevalence of OD has substantially increased since the onset of the coronavirus disease 2019 (COVID-19) pandemic, reaching 30 to 86% of patients infected by *Alpha*, *Delta* or *Omicron* variants [2–4]. The OD may include anosmia, hyposmia or parosmia throughout the clinical course of the disease. According to several studies, the OD may persist in a significant number of patients more than 6-month post-infection and may affect their quality of life (QoL) [4, 5]. The evaluation of OD has to involve psychophysical tests and patient-reported outcome questionnaires that provide additional insight into the impact of OD on patients QoL [6].

In 2005, Frasnelli *et* Hummel developed the Olfactory Disorder Questionnaire (ODQ), which is a patient-reported outcome questionnaire reporting the features of the olfactory disorders and the related impact on QoL [7]. ODQ was validated in English [6], Korean [8] and Chinese [9]. To date, there is no validated version for French speaking countries, which include more than 400 million inhabitants.

## Methods

### Ethical statement

The study protocol was approved by the Institutional Ethics Committee (n° CHUSP20032020)). Patient and healthy individual informed consent was obtained.

### Questionnaire development

A multidisciplinary team composed of two otolaryngologists, a psychologist and a linguist worked on the French adaptation of ODQ (Fr-ODQ) from the English ODQ [6]. Members of the team were native French speakers. Prior to the validation of Fr-ODQ, the draft was sent to 5 patients to detect potential misunderstandings and was improved to have the final version (Additional file 1: Appendix 1).

### Setting

The study was conducted in two medical centers (University of Mons and Dour Medical Center, Dour, Belgium) between January 2021 to April 2022. Irrespective to the etiology, patients with OD were asked to participate to the study. Patients with severe neurological diseases limiting the understanding of the study protocol or those who were not native French-speaker were excluded.

A control group of healthy individuals was composed, matching age and gender of the study group. To be included, healthy individuals had to have no neurological, otolaryngological (sinonasal) or general disorders that may impact the olfactory function. Individuals with a history of COVID-19 were not included in the control group.

### Olfactory and nasal evaluations

Participants completed Fr-ODQ, while nasal complaint evaluation was performed using the French version of the sinonasal outcome tool-22 (SNOT-22) [10, 11]. Psychophysical olfactory evaluations were performed with Threshold, Discrimination and Identification test (TDI; Medisense, Groningen, Netherlands), which is a standardized and validated evaluation of olfaction. The sum of the scores of threshold, discrimination and identification subtests was used clinically to assess olfactory performance. Participants were considered normosmic or dysosmic when TDI ≥ 30.75 and < 30.75, respectively [12].

### Validity, reliability and responsiveness to change

The statistical analyses were performed with Statistical Package for the Social Sciences for Windows (SPSS version 22.0; IBM Corp, Armonk, NY, USA). A level of significance of *p* < 0.05 was used.

The Fr-ODQ was completed twice over 7-day period to evaluate the test–retest reliability (Spearman correlation coefficient). The internal consistency was evaluated with Cronbach’s alpha. External validity was assessed by a correlation analysis between Fr-ODQ and Fr-SNOT-22 (Spearman correlation coefficient). The internal validity was evaluated with a comparison of the Fr-ODQ scores between patients and healthy subjects (Mann–Whitney *U* test.). The responsiveness to change of Fr-ODQ was evaluated in a subgroup of patients with reversible OD, excluding patients with > 2-year post-traumatic loss of smell. Only patients with an improvement of TDI scores 3 months after the initial evaluations were selected for the responsiveness to change analysis. For these patients, authors evaluated the reduction of Fr-ODQ. Patients were invited to adhere to an olfactory training twice daily.

## Results

Eighty-nine patients (67 females) with OD and 65 healthy individuals (50 females) completed the study. The characteristics of patients are reported in Table 1. The most prevalent comorbidities of patients included hypothyroidism, hypertension and hypercholesterolemia. The causes of OD consisted of post-viral (*N* = 80), post-traumatic (*N* = 4), neurological (*N* = 2) and idiopathic (*N* = 3) ODs. The mean duration of OD was 15.7 ± 8.2 months. Parosmia concerned 52 patients (58.4%) patients. Five patients reported TDI > 37 but they suffered from severe parosmia. The mean Fr-SNOT-22 score of patients was 31.80 ± 17.69 (Table 2).

**Table 1:** Epidemiological and clinical outcomes of patients

Outcomes	Patients (*N* = 89)
Age (mean, range)	40.7 ± 13.9 (17–67)
Sex	
Male	22 (24.7)
Female	67 (75.3)
Comorbidities	
Hypothyroidism	14 (15.7)
Hypertension	9 (10.1)
Hypercholesterolemia	9 (10.1)
Tobacco consumption	8 (9.0)
Allergic rhinitis	6 (6.7)
Arthrosis	6 (6.7)
Depression	5 (5.6)
Psoriasis	4 (4.5)
Asthma	4 (4.5)
Diabetes	3 (3.4)
Renal insufficiency	2 (2.2)
Rhumatoid polyarthritis	2 (2.2)
Cancer history	1 (1.1)
Hepatic insufficiency	1 (1.1)
Respiratory insufficiency	1 (1.1)
Cardiologic affections	1 (1.1)
Olfactory dysfunction causes	
Post-viral	80 (89.9)
Post-traumatic	4 (4.5)
Idiopathic	3 (3.4)
Neurological disease	2 (2.2)

**Table 2:** Comparison of Olfactory Questionnaire between patients and healthy individuals

	Olfactory disorders Questionnaire Outcomes	Patients	Controls	*p*-value
	Parosmia outcomes			
P1	Food tastes different than it used to before my accident	2.35 ± 0.93	0.41 ± 0.71	0.001
P2	I can smell something bad, even when other people can’t	1.89 ± 1.11	0.56 ± 0.79	0.001
P3	Some of the smells that I find unpleasant, other people find pleasant	2.08 ± 1.08	0.32 ± 0.67	0.001
P5	Smells smell different to what they used to before my accident	2.08 ± 1.08	0.33 ± 0.68	0.001
	Parosmia total score (/12)	8.23 ± 3.49	1.60 ± 2.06	0.001
	Life Quality Statement Outcomes			
1	I go to restaurants less often than I used to	1.79 ± 1.15	0.63 ± 0.88	0.001
4	I am always aware of the changes in my sense of smell	2.80 ± 0.72	0.33 ± 0.62	0.001
11	I don’t enjoy drinks or food as much as I used to	2.40 ± 0.92	0.58 ± 0.94	0.001
13	I am worried that I will never get used to the changes in my sense of smell	2.14 ± 0.95	0.58 ± 0.90	0.001
15	Because of the changes in my sense of smell, I feel more anxious than I used to feel	1.94 ± 1.02	1.56 ± 1.39	0.001
19	The changes in my sense of smell cause most of my problems	1.42 ± 1.04	0.28 ± 0.58	0.001
22	The changes in my sense of smell annoy me when I am eating	2.24 ± 1.58	0.16 ± 0.57	0.001
26	I visit friends, relatives, or neighbors less often	0.94 ± 1.00	0.17 ± 0.38	0.001
27	Because of the changes in my sense of smell, I try harder to relax	1.46 ± 1.14	1.01 ± 0.90	0.001
28	Because of the changes in my sense of smell I have weight problems	1.71 ± 1.18	0.28 ± 0.58	0.001
32	I can imagine adjusting to the changes in my sense of smell	1.07 ± 1.02	2.70 ± 0.58	0.001
33	The changes in my sense of smell make me feel isolated	1.08 ± 1.06	0.17 ± 0.38	0.001
34	Because of the changes in my sense of smell I avoid groups of people	0.86 ± 0.90	0.16 ± 0.37	0.001
35	The changes in my sense of smell are something I just need to get used to	1.76 ± 0.91	2.50 ± 0.80	0.001
37	I eat less than I used to or more than I used to	1.39 ± 1.10	0.58 ± 0.90	0.001
39	I am scared of getting exposed to certain dangers (e.g., gas, rotten food)	1.85 ± 1.08	0.55 ± 0.85	0.001
42	I have problems with taking part in activities of daily life	0.94 ± 0.85	0.27 ± 0.51	0.001
49	The changes in my sense of smell make me feel angry	1.33 ± 1.06	0.25 ± 0.56	0.001
50	My relationship with my wife / husband / partner is affected	0.89 ± 0.94	0.16 ± 0.37	0.001
	Life Quality Statement Outcomes (/57)	28.35 ± 13.46	5.51 ± 6.76	0.001
	Sincerity Statement Outcomes			
17	Sometimes I have thoughts and ideas I would not want other people to know of	1.24 ± 1.18	0.17 ± 0.42	0.001
31	There are some people who I know that I dislike	0.87 ± 0.92	0.17 ± 0.42	0.001
14	I always keep a promise, no matter what the promise is about or how hard it is for me	1.76 ± 0.91	2.50 ± 0.79	0.001
23	I am always well behaved	1.81 ± 0.96	1.33 ± 1.15	NS
36	I have never been late to an appointment or work	1.82 ± 0.90	1.33 ± 1.14	0.001
48	Sometimes I talk about things I do not understand	1.46 ± 1.13	0.58 ± 0.90	0.001
	Sincerity Statement Score (/18)	8.54 ± 4.44	3.03 ± 1.40	0.001
	Total score (/87)	45.12 ± 18.15	10.13 ± 8.92	0.001
	Scale part: 1 (no annoying) to 10-point (extremely)			
	How annoying the changes in your sense of smell are to you	7.21 ± 3.00	0.53 ± 1.49	0.001
	How often you become aware of the changes to your sense of smell	5.46 ± 3.90	0.27 ± 1.06	0.001
	How severely the changes in your smell affected your professional performance	2.68 ± 2.88	2.48 ± 2.48	0.001
	How severely the changes in your sense of smell affected your recreational activities	1.69 ± 2.71	0.49 ± 1.08	0.001
	How severely the changes in your sense of smell affected your private life	6.74 ± 3.05	0.31 ± 1.15	0.001

*NS* Non-significant

The Cronbach’s alpha value was 0.827 for the items of Fr-ODQ, which indicated a high internal consistency. The test–retest reliability was high for total Fr-ODQ scores (*r*_s_ = 0.944, *p* = 0.001), Parosmia and Life Quality Statement scores and moderate-to-high for Sincerity Statement score (Table 3). The correlation between Fr-ODQ and Fr-SNOT-22 total score was high (*r*_s_ = 0.498; *p* = 0.001), indicating adequate external validity. The mean score of Fr-ODQ was significantly higher in patients compared with controls, which supported an adequate internal validity (Table 4). Seventy-five patients adhered to a 12-week olfactory training protocol for a post-viral OD, consisting of twice daily smell of odor according to the Hummel protocol [13]. The recovery of smell sense occurred after 3.4 ± 4.3 months. Focusing on the 32 patients who had an improvement at the TDI 3 months after the start of the olfactory training, the mean TDI score significantly increased from 19.12 ± 9.08 to 29.50 ± 8.56 (*p* = 0.028); while the Fr-ODQ significantly increased from 45.05 ± 19.25 to 50.82 ± 12.71 (*p* = 0.008), which suggests an adequate responsiveness to change property (Table 4).

**Table 3:** Test re-test reliability findings

	Test–retest	*p*-value
Parosmia score	0.905	0.001
Life Quality Statement score	0.930	0.001
Sincerity Statement score	0.614	0.001
Fr-ODQ total score	0.944	0.001

*Fr-ODQ* French version of olfactory disorder questionnaire

**Table 4:** Responsiveness to Change property

Outcomes	Baseline	3 mo	*p*-value
Parosmia score	7.55 ± 3.65	7.47 ± 3.57	NS
Life Quality Statement score	30.45 ± 14.16	34.25 ± 7.47	0.011
Sincerity Statement score	7.05 ± 4.83	9.00 ± 3.71	0.027
Fr-ODQ total score	45.05 ± 19.25	50.82 ± 12.71	0.008
Threshold	4.27 ± 3.24	6.20 ± 4.63	NS
Discrimination	9.93 ± 3.55	12.17 ± 2.37	0.017
Identification	7.91 ± 4.63	11.29 ± 2.64	0.032
TDI total score	19.12 ± 9.08	29.50 ± 8.56	0.028

The evolution of Fr-ODQ total score was focused on patients who reported psychophysical improvement at the threshold, discrimination, and identification testing

*Fr-ODQ* French version of olfactory disorder questionnaire

## Discussion

The recent increase of the prevalence of OD makes the development of olfactory patient-reported outcome questionnaires important. To date, only the Fr-sQOD-NS was adapted for French speaking countries [14] but this questionnaire only reports limited QoL items. In the present study, we developed a French version of the olfactory disorder questionnaire, which reports high internal consistency and test–retest reliability.

The internal consistency of the Fr-ODQ was comparable with those of the German and English versions. Indeed, the internal consistency of the German version of ODQ was 0.54 and 0.93 for negative and positive statements, respectively [7], while the Cronbach value of the English version of ODQ was 0.90 [6].

The test-restest reliability of the Fr-ODQ was particularly high (*r*_s_ = 0.944) compared with the data of the Frasnelli *et* Hummel version (0.71 and 0.78 for negative and positive statements) [7]. In the study of Langstaff et al*.* the test–retest reliability ranged from 0.56 to 0.77 according to the sub-scores of ODQ [6]. In the present study, the external validity was investigated through a correlation analysis between SNOT-22 and Fr-ODQ. Frasnelli *et* Hummel assessed the external validity through a correlation analysis between Beck Depression Inventory, Mood Inventory and German ODQ questionnaire [7]. They reported significant association between all questionnaires, supporting an adequate external validity. Langstaff et al*.* did not find significant external validity comparing TDI and ODQ scores [6]. The use of SNOT-22 in the present study limits us in the comparison of our results with those of the literature. We have chosen SNOT-22 because that was the closest questionnaire to the ODQ regarding symptoms and QoL impact. Indeed, there is no other olfactory questionnaire validated in French at the exception of Fr-sQOD-NS [14], which included items of the ODQ. The high internal validity outcomes of the Fr-ODQ corroborated those of the German version in which Frasnelli et Hummel observed significant higher score of ODQ in hyposmia/anosmic patients compared with normosmic individuals [7].

Responsiveness to change is another important parameter in the validation of a patient-reported outcome questionnaire [15]. The originality of the validation of the Fr-ODQ was the assessment of the ‘responsiveness to change’ property. Focusing on patients who reported improvement at the TDI scores (at least 1 point increase), we observed a similar significant improvement of Fr-ODQ total score, suggesting an adequate ‘responsiveness to change’ property.

The primary limitation of this study are the low number of patients and the high proportion of post-viral OD individuals. The characteristics of patients with post-viral OD may be different from the OD of patients with chronic rhinosinusitis with nasal polyps or other etiologies. The high proportion of post-viral OD was related to the onset of the COVID-19 pandemic and the increase of the number of anosmic, hyposmic and parosmic patients in the general population. The German and English version of ODQ were both validated before the pandemic and, therefore, included more patients with non-COVID-19 OD. In future studies, authors may use the smell identification test (UPSIT) in addition to TDI to strengthen the olfactory evaluation. The Fr-ODQ could be used in many future French studies from France, Belgium or Canada in which authors will investigate smell function in common conditions, including chronic rhinosinusitis with or without polyps [16, 17], or COVID-19 [18]. The main strengths of this study are the realization of TDI evaluations, allowing the confirmation of the olfactory dysfunction and the evaluation of ‘responsiveness to change’ parameter.

## Conclusion

The Fr-ODQ is the first patient-reported outcome questionnaire validated for French speaking patients. Fr-ODQ is reliable and valid for the evaluation of the olfactory dysfunction and the related impact on QoL of French-speaking patients.

## Supplementary Information

**Additional file 1. Appendix 1:** The French version of Olfactory Disorder Questionnaire.

## Data Availability

Available on request.
