# Supplementary material for: Validity and reliability of a french version of the olfactory disorders questionnaire
Source: J Otolaryngol Head Neck Surg. 2022 Oct 1;51:36. doi: 10.1186/s40463-022-00598-2 (PMC9526523; doi:10.1186/s40463-022-00598-2)
Supplement: Supplementary file 1 — Additional file 1. Appendix 1: The French version of Olfactory Disorder Questionnaire. [file 40463_2022_598_MOESM1_ESM.docx]

**Appendix 1: The French version of Olfactory Disorder Questionnaire.**

Partie 1: Parosmie. Veuillez entourer la bonne réponse.

| P1 | La nourriture a changé de goût depuis mon accident | Entièrement d’accord |  |
| --- | --- | --- | --- |
|  |  | Partiellement d’accord |  |
|  |  | Partiellement en désaccord |  |
|  |  | Entièrement en désaccord |  |
| P2 | Il m’arrive de sentir de mauvaises odeurs, même quand les personnes autour ne les sentent pas. | Entièrement d’accord |  |
|  |  | Partiellement d’accord |  |
|  |  | Partiellement en désaccord |  |
|  |  | Entièrement en désaccord |  |
| P3 | Certaines odeurs que je trouve déplaisantes sont agréables pour les autres | Entièrement d’accord |  |
|  |  | Partiellement d’accord |  |
|  |  | Partiellement en désaccord |  |
|  |  | Entièrement en désaccord |  |
| P5 | Un de mes plus gros problèmes est que les odeurs me semblent différentes depuis mon accident | Entièrement d’accord |  |
|  |  | Partiellement d’accord |  |
|  |  | Partiellement en désaccord |  |
|  |  | Entièrement en désaccord |  |

Partie 2 : Life Quality Statement outcomes.

| 1 | En raison des changements qui affectent mon odorat, je vais moins souvent au restaurant | Entièrement d’accord |  |
| --- | --- | --- | --- |
|  |  | Partiellement d’accord |  |
|  |  | Partiellement en désaccord |  |
|  |  | Entièrement en désaccord |  |
| 4 | Je m’aperçois toujours des changements dans mon odorat | Entièrement d’accord |  |
|  |  | Partiellement d’accord |  |
|  |  | Partiellement en désaccord |  |
|  |  | Entièrement en désaccord |  |
| 11 | En raison des changements qui affectent mon odorat, je n’apprécie plus autant la nourriture et les boissons qu’avant | Entièrement d’accord |  |
|  |  | Partiellement d’accord |  |
|  |  | Partiellement en désaccord |  |
|  |  | Entièrement en désaccord |  |
| 13 | Je suis inquiet de ne jamais m’habituer aux changements de mon odorat | Entièrement d’accord |  |
|  |  | Partiellement d’accord |  |
|  |  | Partiellement en désaccord |  |
|  |  | Entièrement en désaccord |  |
| 14 | Je tiens toujours une promesse, quelle que soit sa nature ou la difficulté que j’ai à la respecter | Entièrement d’accord |  |
|  |  | Partiellement d’accord |  |
|  |  | Partiellement en désaccord |  |
|  |  | Entièrement en désaccord |  |
| 15 | En raison des changements qui affectent mon odorat, je me sens plus anxieux qu’avant | Entièrement d’accord |  |
|  |  | Partiellement d’accord |  |
|  |  | Partiellement en désaccord |  |
|  |  | Entièrement en désaccord |  |
| 17 | Je n’aimerais pas que certaines de mes pensées soient connues des autres | Entièrement d’accord |  |
|  |  | Partiellement d’accord |  |
|  |  | Partiellement en désaccord |  |
|  |  | Entièrement en désaccord |  |
| 19 | La majorité de mes problèmes sont causés par les changements affectant mon odorat | Entièrement d’accord |  |
|  |  | Partiellement d’accord |  |
|  |  | Partiellement en désaccord |  |
|  |  | Entièrement en désaccord |  |
| 22 | Les changements dans mon odorat me dérangent lorsque je mange | Entièrement d’accord |  |
|  |  | Partiellement d’accord |  |
|  |  | Partiellement en désaccord |  |
|  |  | Entièrement en désaccord |  |
| 23 | Je me comporte toujours bien | Entièrement d’accord |  |
|  |  | Partiellement d’accord |  |
|  |  | Partiellement en désaccord |  |
|  |  | Entièrement en désaccord |  |
| 26 | En raison des changements qui affectent mon odorat, je visite moins souvent mes amis, ma famille et mon entourage | Entièrement d’accord |  |
|  |  | Partiellement d’accord |  |
|  |  | Partiellement en désaccord |  |
|  |  | Entièrement en désaccord |  |
| 27 | En raison des changements qui affectent mon odorat, j’essaie de me détendre davantage. | Entièrement d’accord |  |
|  |  | Partiellement d’accord |  |
|  |  | Partiellement en désaccord |  |
|  |  | Entièrement en désaccord |  |
| 28 | En raison des changements qui affectent mon odorat, j’ai des problèmes de poids | Entièrement d’accord |  |
|  |  | Partiellement d’accord |  |
|  |  | Partiellement en désaccord |  |
|  |  | Entièrement en désaccord |  |

| 31 | Je ne supporte pas certaines des personnes que je connais. | Entièrement d’accord |  |
| --- | --- | --- | --- |
|  |  | Partiellement d’accord |  |
|  |  | Partiellement en désaccord |  |
|  |  | Entièrement en désaccord |  |
| 32 | Je pense être en mesure de m’adapter aux changements dans mon odorat | Entièrement d’accord |  |
|  |  | Partiellement d’accord |  |
|  |  | Partiellement en désaccord |  |
|  |  | Entièrement en désaccord |  |
| 33 | Je me sens isolé en raison des changements de mon odorat | Entièrement d’accord |  |
|  |  | Partiellement d’accord |  |
|  |  | Partiellement en désaccord |  |
|  |  | Entièrement en désaccord |  |
| 34 | En raison des changements qui affectent mon odorat, j’évite les groupes de personnes | Entièrement d’accord |  |
|  |  | Partiellement d’accord |  |
|  |  | Partiellement en désaccord |  |
|  |  | Entièrement en désaccord |  |
| 35 | Je dois simplement m’habituer aux changements qui affectent mon odorat. | Entièrement d’accord |  |
|  |  | Partiellement d’accord |  |
|  |  | Partiellement en désaccord |  |
|  |  | Entièrement en désaccord |  |
| 36 | Je n’ai jamais été en retard à un rendez-vous ou au travail | Entièrement d’accord |  |
|  |  | Partiellement d’accord |  |
|  |  | Partiellement en désaccord |  |
|  |  | Entièrement en désaccord |  |
| 37 | En raison des changements qui affectent mon odorat, je mange plus ou moins qu'avant | Entièrement d’accord |  |
|  |  | Partiellement d’accord |  |
|  |  | Partiellement en désaccord |  |
|  |  | Entièrement en désaccord |  |
| 39 | En raison de la dysfonction de mon odorat, j’ai peur d’être exposé à certains dangers (exemples: fuite de gaz, nourriture pourrie) | Entièrement d’accord |  |
|  |  | Partiellement d’accord |  |
|  |  | Partiellement en désaccord |  |
|  |  | Entièrement en désaccord |  |
| 42 | En raison des changements dans mon odorat, j’ai des difficultés à effectuer mes activités quotidiennes | Entièrement d’accord |  |
|  |  | Partiellement d’accord |  |
|  |  | Partiellement en désaccord |  |
|  |  | Entièrement en désaccord |  |
| 48 | Je parle parfois de chose que je ne comprends pas | Entièrement d’accord |  |
|  |  | Partiellement d’accord |  |
|  |  | Partiellement en désaccord |  |
|  |  | Entièrement en désaccord |  |
| 49 | Les changements dans mon odorat me mettent en colère | Entièrement d’accord |  |
|  |  | Partiellement d’accord |  |
|  |  | Partiellement en désaccord |  |
|  |  | Entièrement en désaccord |  |
| 50 | En raison des changements qui affectent mon odorat, ma relation avec ma femme / mon mari / mon partenaire est affectée | Entièrement d’accord |  |
|  |  | Partiellement d’accord |  |
|  |  | Partiellement en désaccord |  |
|  |  | Entièrement en désaccord |  |

À l’aide de l’échelle suivante, indiquez à quel point les changements dans votre odorat vous dérangent.

Pas du tout très dérangeants

dérangeants

À l’aide de l’échelle suivante, indiquez à quelle fréquence vous prenez conscience de changements dans votre odorat

jamais très fréquemment

À l’aide de l’échelle suivante, indiquez à quel point l’altération de votre odorat a affecté votre vie professionnelle durant le dernier mois.

Pas du tout énormément

À l’aide de l’échelle suivante, indiquez à quel point l’altération de votre odorat a affecté vos activités récréatives (par exemple vos passe-temps et vos intérêts) dans le dernier mois.

Pas du tout énormément

À l’aide de l’échelle suivante, indiquez à quel point l’altération de votre odorat a affecté votre vie privée dans le dernier mois.

Pas du tout énormément

**Appendix 1 footnotes:** The Questionnaire of Olfactory Disorders (QOD) consists of two parts: 1) Statements and 2) Visual Analogue Scales.

## Statements

There are three different kinds of statements: Each statement has to be answered by checking the box of the appropriate answer: I agree, I partly agree, I partly disagree, and I disagree. Patients have to be advised not to miss any of the statements and to work as fast as possible, without wasting too much time at a single statement.

### “Life Quality statements“

All statements without those described under 1.2. and 1.3. are Life Quality statements. They express the patients’ complaints related to the difficulties with smelling. For checking the box “agree” 3 points are assigned; 2 points are assigned for “agree partly”, 1 point is assigned for “disagree partly”, 0 points are assigned for “disagree”. Assignment is inverse for the statements 32 and 35 (positive statements): “agree”: 0 points, “partly agree”: 1 point, “partly disagree”: 2 points, “disagree”: 3 points.

The sum of the scores is the Life Quality raw score (LQ_rv_); maximal 57 points can be reached. LQ_rv_ is transformed into the Life Quality score (LQ) by the formula

LQ=LQ_rv_/0.57 [%].

High scores indicate a strong impairment.

Life Quality statements: 1, 4, 11, 13, 15, 19, 22, 26, 27, 28, 33, 34, 37, 39, 42, 49, 50; positive statements: 32, 35.

### “Sincerity statements“

A tendency toward giving socially desired answers can be assessed by means of the Sincerity statements. At statements 17, 31, and 48, for checking the box “agree” 3 points are assigned; 2 points are assigned for “agree partly”, 1 point is assigned for “disagree partly”, 0 points are assigned for “disagree”. Assignment is inverse for the statements 14, 23, and 36: “agree”: 0 points, “partly agree”: 1 point, “partly disagree”: 2 points, “disagree”: 3 points. Sum of the scores makes the Sincerity raw score (S_rv_). A maximal score of 18 can be reached. S_rv_ is transformed into the sincerity score (S) by the formula

S=S_rv_/0.18 [%].

Low scores indicate a tendency towards giving socially desired answers.

Sincerity statements: 14, 17, 23, 31, 36, 48.

### “Parosmia statements”

Parosmia is assessed by means of the Parosmia statements. Points are assigned in analogy to 1.1. Maximal Parosmia raw score (P_rv_) is 12. Transformation follows the formula

P=P_rv_/0.12 [%].

High scores indicate parosmia.

Parosmia statements: P1, P2, P3, P5.

## Scales

In the scales part difficulties with smelling are assessed by means of five visual analogue scales. They regard how annoying the difficulties are (1), how often patients become aware of them (2), how strong patients are affected in the job (3), in their free time (4), and in their private life (5).
